# Effects of lymphocyte and neutrophil counts and their time courses on mortality in patients with postoperative pneumonia

**DOI:** 10.1038/s41598-022-18794-5

**Published:** 2022-08-26

**Authors:** Yasushi Murakami, Yuichiro Shindo, Masahiro Sano, Junya Okumura, Hironori Kobayashi, Toshihiro Sakakibara, Mitsutaka Iguchi, Kunihiko Takahashi, Tetsuya Yagi, Shigeyuki Matsui, Yoshinori Hasegawa

**Affiliations:** 1 Department of Respiratory Medicine, Nagoya University Graduate School of Medicine, 65 Tsurumai-cho, Showa-ku, Nagoya, 466-8550 Japan; 2 Department of Respiratory Medicine, National Hospital Organization Higashinagoya National Hospital, Nagoya, Japan; 3 Department of Respiratory Medicine, Toyota Memorial Hospital, Toyota, Japan; 4 Department of Infectious Diseases, Nagoya University Hospital, Nagoya, Japan; 5 Department of Biostatistics, Nagoya University Graduate School of Medicine, Nagoya, Japan; 6 Department of Biostatistics, M&D Data Science Center, Tokyo Medical and Dental University, Tokyo, Japan; 7 National Hospital Organization, Nagoya Medical Center, Nagoya, Japan

**Keywords:** Infectious diseases, Respiratory tract diseases

## Abstract

The prognostic significance of absolute lymphocyte count (ALC) and absolute neutrophil count (ANC) remains unclear in patients with postoperative pneumonia (POP). The study objectives were to investigate the prognostic effects of ALC and ANC in POP patients, and to evaluate the time courses of ALC and ANC during hospitalization. This post-hoc analysis of a single-center prospective observational study evaluated consecutive POP patients, and comparatively analyzed community-acquired pneumonia (CAP) patients to highlight features of POP. In total, 228 POP patients and 1027 CAP patients were assessed. Severe lymphopenia (ALC < 500 cells/μL) at diagnosis was associated with worse 90-day survival in both types of pneumonia. In POP patients, neutrophilia (ANC > 7500 cells/μL) was associated with better survival, whereas CAP patients with neutrophilia tended to have a lower survival rate. Prolonged lymphopenia and delayed increase in neutrophils were characteristic time-course changes of non-survivors in POP. The time courses of ALC and ANC between survivors and non-survivors in POP trended differently from those in CAP. Our study showed that ALC and ANC at pneumonia diagnosis can serve as prognostic factors in POP patients. Differences in time-course changes of ALC and ANC between survivors and non-survivors may provide important information for future immunological research in pneumonia.

## Introduction

Postoperative pneumonia (POP) is a serious infectious complication associated with increased morbidity and mortality in postoperative patients^1–6^. Despite advances in surgical techniques, anesthesia, and perioperative care, POP remains one of the most common postoperative complications^7^. The mortality in patients with POP even when appropriate antimicrobials are prescribed is high^1–3^. Thus, a better understanding of the state of illness and novel management strategies are needed to improve POP outcomes.

It is important to assess individual risk factors associated with mortality to optimize treatment for patients with pneumonia, and numerous prognostic indicators have been established^8,9^. Among these factors, the absolute lymphocyte count (ALC) and absolute neutrophil count (ANC) in blood have been recently recognized as useful risk stratification markers for patients with pneumonia^10–13^. Previous studies in patients with community-acquired pneumonia (CAP) have shown that lymphopenia or failure to increase the neutrophil count at diagnosis was associated with an increased mortality risk^11,13^. The neutrophil to lymphocyte ratio (NLR) has also been reported as a risk factor for mortality in patients with CAP^10^. Furthermore, ALC and ANC can potentially provide information about individual immunological phenotypes of patients with pneumonia. Specifically, lymphopenia is a marker of impaired immunity in critically ill patients, and an increased number of circulating neutrophils usually reflects systemic inflammation^14–17^. Therefore, investigation of the relationship between these immune cell counts and mortality would be helpful to characterize individual host immunological phenotypes associated with poor outcomes in patients with pneumonia; however, there is a lack of evidence regarding patients with POP in the literature. POP is a second-hit infection following a surgical insult and is associated with postoperative immune dysregulation^18,19^. Thus, investigating the kinetics of ALC and ANC in patients with POP may highlight the immunological features that are distinct from those of CAP.

The study aim was to investigate the prognostic effects of ALC and ANC in patients with POP. Furthermore, we evaluated the time courses of ALC and ANC during hospitalization. To highlight features of POP as a second-hit infection, we comparatively analyzed patients with CAP.

## Methods

### Study design and participants

This study was a post-hoc analysis of a prospective observational study conducted between March 2010 and March 2017 at Nagoya University Hospital (a tertiary hospital, Nagoya, Japan). Details of the study methods have been described elsewhere^20^. Briefly, patients aged ≥ 20 years old with pneumonia who needed in-hospital treatment at the study institution were consecutively enrolled. Pneumonia was diagnosed according to international guidelines^21,22^, and was classified into the following three categories: ventilator-associated pneumonia (VAP), hospital-acquired pneumonia (HAP), and CAP, including health-care associated pneumonia (HCAP). These definitions have been described previously^23^. All patients received antimicrobials at the discretion of the physicians in charge. This study was approved by the Ethics Review Committee of Nagoya University School of Medicine and adhered to the Japanese Ethical Guidelines for Medical and Health Research Involving Human Subjects. The Ethics Review Committee of Nagoya University School of Medicine approved that informed consent of the participants was waived, but the opt-out method was adopted in accordance with the Japanese ethics guidelines. This study was registered with the University Hospital Medical Information Network in Japan (number, UMIN000007090).

Postoperative pneumonia was included in HAP or VAP and defined as pneumonia that developed ≤ 30 days after surgery^5^. Patients with POP were analyzed in this study. To highlight features of POP as a second-hit infection, we comparatively analyzed patients with CAP as a first-hit infection. The patients were excluded from analyses when they met the following criteria: had undergone surgery under topical anesthesia, had an immunocompromised disorder (human immunodeficiency virus-positive, hematological diseases, primary immunodeficiency diseases, asplenia, solid-organ transplantation, ≥ 14 days of treatment with ≥ 10 mg/day of prednisone or equivalent, cytotoxic chemotherapy, and other immunosuppressive drugs), and had missing data of ALC or ANC at pneumonia diagnosis. When patients had multiple registrations, only data from the initial admission were evaluated.

### Data collection and definition of variables

Data prospectively collected at pneumonia diagnosis (day 0) included baseline demographic information, laboratory findings at diagnosis, illness severity, microbiological characteristics, and initial treatment for pneumonia. Clinical outcome data were collected 30 and 90 days after the diagnosis of pneumonia. Detailed information on the preceding surgical procedures and sequential laboratory findings, including total and differential leukocyte counts, were retrospectively collected from medical records. For patients with POP, to estimate the severity of surgical procedures, the surgery severity score was calculated using the Physiological and Operative Severity Score for enUmeration of Mortality and morbidity (POSSUM), which is a scoring system for prediction of perioperative mortality and morbidity in various types of surgery^24^. Details of the microbiological evaluation have been described elsewhere^20^. Antibiotic treatment was classified as appropriate when the identified pathogens were susceptible to the initially prescribed antibiotic(s)^23^.

Total and differential leukocyte counts were submitted for routine clinical analysis in the laboratories of the study institution. ALC and ANC were evaluated at the following time points: day 0–1 (day 0), day 2–4 (day 3), day 5–9 (day 7), day 11–17 (day 14), day 18–24 (day 21), and day 25–31 (day 28). For patients with POP, ALC, and ANC before pneumonia diagnosis were additionally assessed (i.e., findings of the preoperative laboratory test and the first and last postoperative laboratory test before diagnosis of POP).

### Outcome measures

The primary endpoint was 90-day all-cause mortality, defined as death within 90 days after pneumonia diagnosis. In addition, 90-day pneumonia-related mortality was assessed. Pneumonia-related mortality was defined as death caused by respiratory failure or multiple organ failure following POP. This cause of death was confirmed by the physician in charge and one of the research members (YM, MS, JO, TS, HK, and YS).

### Statistical analysis

All statistical tests were two-tailed, and a *p* value of < 0.05 was considered indicative of statistical significance. Categorical data were described as frequencies in percentage, and continuous data were described as the median with interquartile range. Pearson’s chi-square test or Fisher’s exact test was used to analyze discrete variables, as appropriate, and the Mann–Whitney U-test was used to analyze continuous variables.

To assess the effects of ALC and ANC on 90-day all-cause mortality, we performed survival analysis based on ALC and ANC at the diagnosis of pneumonia. In this analysis, ALC and ANC were categorized into the following classes considering previous literatures and the upper limit of the normal range of ANC: without lymphopenia (ALC ≥ 1 × 10^3^ cells/μL), mild lymphopenia (0.5 × 10^3^ cells/μL ≤ ALC < 1 × 10^3^ cells/μL), and severe lymphopenia (ALC < 0.5 × 10^3^ cells/μL)^11,16,25^. ANC was categorized as follows: with neutrophilia (ANC > 7.5 × 10^3^ cells/μL) and without neutrophilia (ANC ≤ 7.5 × 10^3^ cells/μL). The Kaplan–Meier method was used to generate survival curves, and the log-rank test was performed for comparisons among different groups. Time to death due to any cause was estimated from the date of diagnosis of pneumonia. The Cox proportional hazards model was used to estimate the hazard ratio (HR) and 95% confidence interval (CI) of ALC and ANC for 90-day mortality. Several clinically important confounders, i.e., age, sex, severity of illness [surgery severity score and the Sequential Organ Failure Assessment (SOFA) score in patients with POP and the Pneumonia Severity Index in patients with CAP] were included in the multivariable models.

As sub-analyses, the effects of ALC and ANC on 90-day pneumonia-related mortality were assessed using the Cox proportional hazards model. Furthermore, among patients who received appropriate initial antibiotic treatment, the Kaplan–Meier survival curves were generated, and the log-rank test was performed. In addition, the effects of ALC and ANC at diagnosis of pneumonia on 90-day all-cause and pneumonia-related mortality in those patients were assessed, as well as the above main analysis. All statistical data were analyzed using SPSS Statistics software (version 26; IBM, Armonk, NY, USA).

## Results

### Participants and baseline characteristics

**Figure 1:**
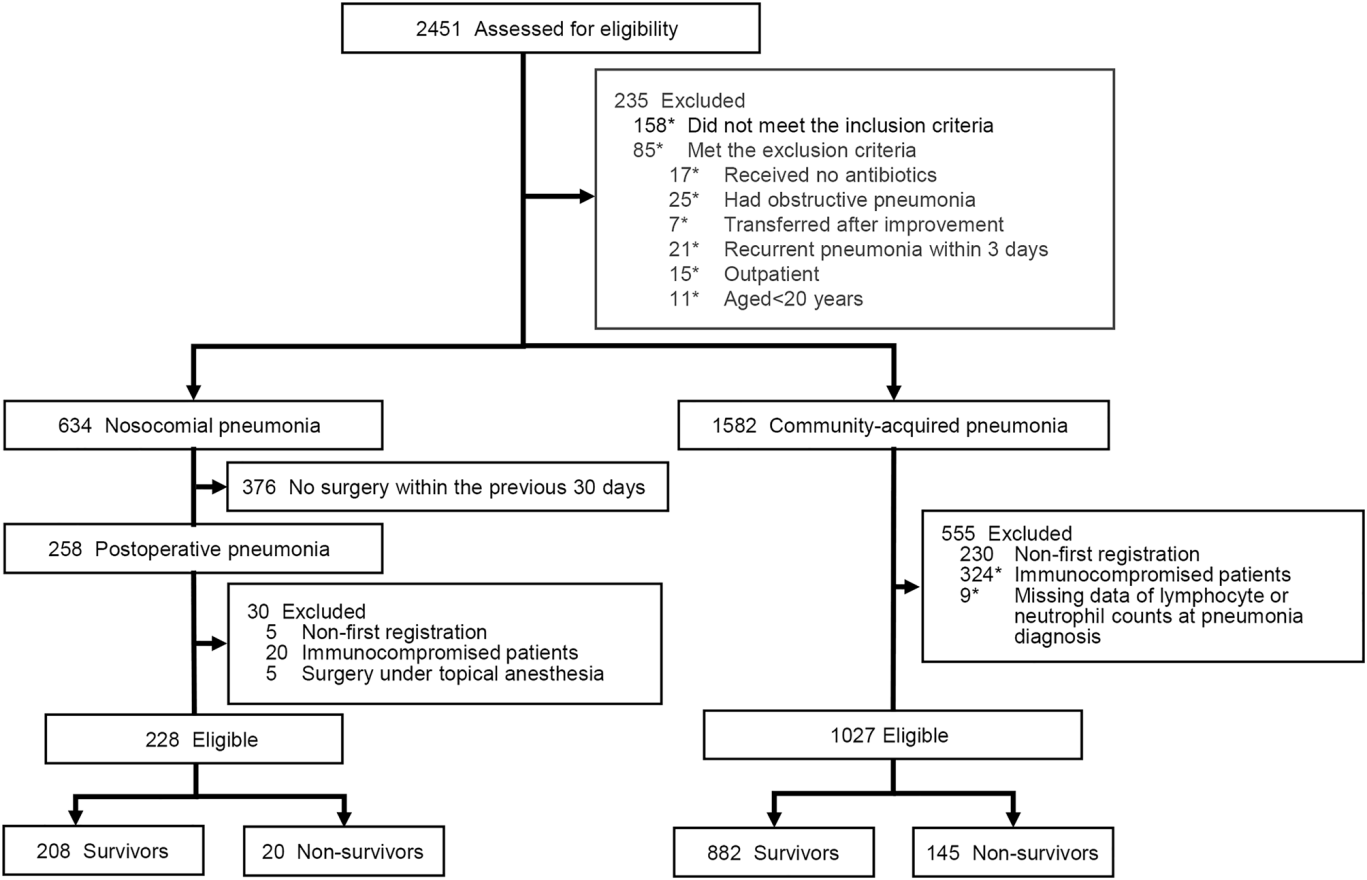
Patient flow. *Included overlapping cases.

Of 2451 patients assessed for eligibility, 634 had nosocomial pneumonia and 1582 had CAP. Among these 634 and 1582 patients, 228 with POP and 1027 with CAP without immunocompromised disorders were included in the analysis (Fig. 1). In the patients with POP and CAP, 20 (8.8%) and 145 (14.1%) died within 90 days after diagnosis of pneumonia, respectively. Of the patients with POP, 12 died due to pneumonia-related death.

Table 1 shows the characteristics in patient with POP and CAP. In the patients with POP, there were no differences in age, sex, frequency of VAP, low body weight, and comorbidities between survivors and non-survivors. The median time to pneumonia diagnosis from surgery was 5 days in the survivors and non-survivors. Although the frequency of gastroenterological and lung surgery was higher in the non-survivors, the estimated surgery severity scores were comparable between survivors and non-survivors. The SOFA score and frequency of low blood pressure tended to be higher in the non-survivors. In the patients with CAP, there were significant differences in age, sex, frequency of low body weight and several comorbidities, and severity of illness between the survivors and non-survivors. Causative pathogens were identified in 155 patients with POP (68.0%) and 441 with CAP (42.9%). Among the patients with POP, *Enterobacter* spp. were most frequent, followed by *Pseudomonas aeruginosa* and *Klebsiella pneumoniae*. The details are shown in Supplementary Table S1. Among the POP patients with identified pathogens, appropriate initial antibiotics were administered in 88 (62.9%) of 140 survivors and 13 (86.7%) of 15 non-survivors. Less than 5% of patients received corticosteroids for treatment of pneumonia in both types of pneumonia.

**Table 1:** Patient characteristics in postoperative pneumonia and community-acquired pneumonia.

	Postoperative pneumonia				Community-acquired pneumonia			
	Total	Survivors	Non-survivors	*P* value	Total	Survivors	Non-survivors	*P* value
	(n = 228)	(n = 208)	(n = 20)		(n = 1027)	(n = 882)	(n = 145)	
**Baseline characteristics at pneumonia diagnosis**								
Age, median (IQR)	70 (64–76)	70 (64–76)	70 (68–76)	0.901	75 (67–83)	75 (66–82)	78 (70–86)	0.001
Female	35 (15.4)	30 (14.4)	5 (25.0)	0.173	332 (32.3)	302 (34.2)	30 (20.7)	0.001
Ventilator-associated pneumonia	65 (28.5)	59 (28.4)	6 (30.0)	0.877	–	–	–	–
Body mass index < 18.5 kg/m^2^*	55 (24.1)	50 (24.0)	5 (25.0)	0.555	269 (30.4)	224 (28.8)	45 (41.7)	0.006
**Comorbidities**								
Neoplastic diseases†	129 (56.6)	114 (54.8)	15 (75.0)	0.082	279 (27.2)	228 (25.9)	51 (35.2)	0.019
Congestive heart failure	63 (27.6)	59 (28.4)	4 (20.0)	0.424	165 (16.1)	135 (15.3)	30 (20.7)	0.102
Chronic lung diseases	48 (21.1)	41 (19.7)	7 (35.0)	0.098	306 (29.8)	250 (28.3)	56 (38.6)	0.012
Chronic renal failure	36 (15.8)	33 (15.9)	3 (15.0)	0.609	132 (12.9)	109 (12.4)	23 (15.9)	0.243
Chronic liver diseases	8 (3.5)	6 (2.9)	2 (10.0)	0.149	43 (4.2)	34 (3.9)	9 (6.2)	0.190
Diabetes	43 (18.9)	38 (18.3)	5 (25.0)	0.317	236 (23)	198 (22.4)	38 (26.2)	0.319
**Precedent surgery**								
Time to pneumonia diagnosis from surgery, days, median (IQR)	5 (3–10)	5 (3–10)	5 (3–12)	0.923	–	–	–	–
**Type of surgery**				0.015				
Cardiovascular surgery	85 (37.3)	81 (38.9)	4 (20.0)		–	–	–	–
Gastroenterological surgery	79 (34.6)	68 (32.7)	11 (55.0)		–	–	–	–
Lung surgery	31 (13.6)	26 (12.5)	5 (25.0)		–	–	–	–
Other surgery‡	33 (14.5)	33 (15.9)	0		–	–	–	–
Surgery severity score§, median (IQR)	16 (13–20)	16 (13–20)	18 (15–22)	0.105	–	–	–	–
**Severity of illness**								
SOFA score at pneumonia diagnosis, median (IQR)	4 (2–9)	4 (2–9)	8 (4–11)	0.091	3 (1–4)	2 (1–4)	4 (2–5)	< 0.001
Low blood pressure^||^	131 (57.5)	116 (55.8)	15 (75.0)	0.097	298 (29)	253 (28.7)	45 (31.0)	0.563
**Pneumonia severity index class**								< 0.001
I–III	–	–	–	–	450 (43.8)	419 (47.5)	31 (21.4)	
IV	–	–	–	–	326 (31.7)	275 (31.2)	51 (35.2)	
V	–	–	–	–	251 (24.4)	188 (21.3)	63 (43.4)	
Causative pathogens were identified^¶^	155 (68.0)	140 (67.3)	15 (75.0)	0.481	441 (42.9)	380 (43.1)	61 (42.1)	0.819
Appropriate initial antibiotic treatment	101/155 (65.2)	88/140 (62.9)	13/15 (86.7)	0.066	–	–	–	–
**Treatment of pneumonia**								
Corticosteroid for treatment of pneumonia	4 (1.8)	1 (0.5)	3 (15.0)	0.002	31 (3)	28 (3.2)	3 (2.1)	0.471

*VAP* entilator-associated pneumonia, *BMI* Body mass index, *OSS* Operative severity score, *SOFA* Sequential organ failure assessment, *PSI* Pneumonia severity index, *IQR* Interquartile range.

Data are presented as n (%) unless indicated otherwise.

*Body mass index was assessed in all patients with postoperative pneumonia, 886 patients (86.3%) with community-acquired pneumonia (778 survivors, and 108 non-survivors).

^†^Neoplastic diseases were defined as any cancer that was active at the time of presentation or diagnosed within 5 years of presentation.

^‡^Intracranial surgery (8 patients), head and neck surgery (16 patients), urological surgery (4 patients), orthopedic surgery (4 patients), and dermatological surgery (1 patients) were included.

^§^Score was calculated using the physiological and operative severity score for the enumeration of the mortality and morbidity (POSSUM) scoring system.

^||^Systolic blood pressure < 90 mmHg or diastolic blood pressure ≤ 60 mmHg or use of vasopressor.

^¶^Major pathogens were as follows: *Enterobacter* spp. (39 (17.1%)), *Pseudomonas aeruginosa* (37 (16.2%)), and *Klebsiella pneumoniae* (28 (12.3%)) in POP, and *Staphylococcus aureus* (87 (8.5%)), *K. pneumoniae* (83 (8.1%)), and *Streptococcus pneumoniae* (70 (6.8%)) in CAP.

### Laboratory findings of lymphocytes and neutrophils at pneumonia diagnosis

Laboratory findings of lymphocytes and neutrophils at pneumonia diagnosis are summarized in Table 2. In the patients with POP, 65.8% of the total patients had lymphopenia, and non-survivors had more severe lymphopenia than survivors. ANC was lower for the non-survivors than for the survivors. Neutrophilia was found in only 40.0% of the non-survivors but in 68.3% of the survivors. The percentages of lymphocytes and neutrophils among the total leukocytes and NLR did not differ between the survivors and non-survivors. Lymphopenia was observed in 53.7% of all patients with CAP, and ALC was lower in the non-survivors than survivors. Over 60% of the survivors and non-survivors showed neutrophilia. Non-survivors had higher ANC than survivors. Compared with survivors, non-survivors had a lower percentage of ALC in the total leukocytes and higher percentages of ANC and NLR.

**Table 2:** Findings of blood leukocytes, lymphocytes, and neutrophils at pneumonia diagnosis.

	Postoperative pneumonia				Community-acquired pneumonia			
	Total	Survivors	Non-survivors	*P* value	Total	Survivors	Non-survivors	*P* value
	(n = 228)	(n = 208)	(n = 20)		(n = 1027)	(n = 882)	(n = 145)	
**Absolute count**								
Leukocyte, × 10^3^/μL, median (IQR)	11.1 (8.2–15.6)	11.2 (8.4–15.8)	8.2 (3.0–12.5)	0.013	10.9 (8.2–14.0)	10.8 (8.1–13.8)	11.7 (8.6–15.3)	0.049
Lymphocyte, × 10^3^/μL, median (IQR)	0.8 (0.6–1.1)	0.8 (0.6–1.1)	0.7 (0.3–0.9)	0.035	0.9 (0.6–1.4)	0.9 (0.6–1.4)	0.8 (0.5–1.2)	0.003
Without lymphopenia*, n (%)	78 (34.2)	74 (35.6)	4 (20.0)	0.015	476 (46.3)	422 (47.8)	54 (37.2)	0.018
Mild lymphopenia*, n (%)	111 (48.7)	103 (49.5)	8 (40.0)		420 (40.9)	356 (40.4)	64 (44.1)	
Severe lymphopenia*, n (%)	39 (17.1)	31 (14.9)	8 (40.0)		131 (12.8)	104 (11.8)	27 (18.6)	
Neutrophil, × 10^3^/μL, median (IQR)	9.4 (6.8–13.5)	9.5 (7.0–13.5)	7.0 (2.5–11.4)	0.025	9.1 (6.4–12.0)	8.9 (6.3–11.9)	9.9 (7.0–13.6)	0.010
With neutrophilia^†^, n (%)	150 (65.8)	142 (68.3)	8 (40.0)	0.011	653 (63.6)	552 (62.6)	101 (69.7)	0.101
**Percentage‡**								
Lymphocyte, %, median (IQR)	7.4 (5.1–10.7)	7.2 (5.1–10.7)	9.3 (5.4–13.5)	0.276	9.0 (5.5–13.8)	9.1 (5.7–14.3)	7.1 (4.2–11.5)	< 0.001
Neutrophil, %, median (IQR)	85.3 (80.2–89.3)	85.5 (80.1–89.3)	84.8 (81.8–87.7)	0.739	84.3 (77.7–89.5)	83.7 (77.2–89.3)	88.0 (81.2–91.3)	< 0.001
NLR, median (IQR)	11.5 (7.6–17.5)	11.7 (7.7–17.5)	9.2 (5.9–17.2)	0.363	9.3 (5.6–16.4)	9.0 (5.5–15.5)	12.4 (7.4–22.3)	< 0.001

*IQR* Interquartile range, *NLR* Neutrophil to lymphocyte ratio.

*Patients were classified according to absolute lymphocyte counts (ALCs) at pneumonia diagnosis, as follows: severe lymphopenia, ALC < 0.5 × 10^3^ cells/μL; mild lymphopenia, ALC of 0.5–0.9 × 10^3^ cells/μL; and without lymphopenia, ALC ≥ 1.0 × 10^3^ cells/μL.

^†^According to absolute neutrophil counts (ANCs) at pneumonia diagnosis, patients with ANC > 7.5 × 10^3^ cells/μL were defined as those with neutrophilia and patients with ANC ≤ 7.5 × 10^3^ cells/μL were defined as those without neutrophilia.

^‡^Percentages indicate those among total leukocytes.

### Effects of lymphocyte and neutrophil counts at pneumonia diagnosis on mortality

**Figure 2:**
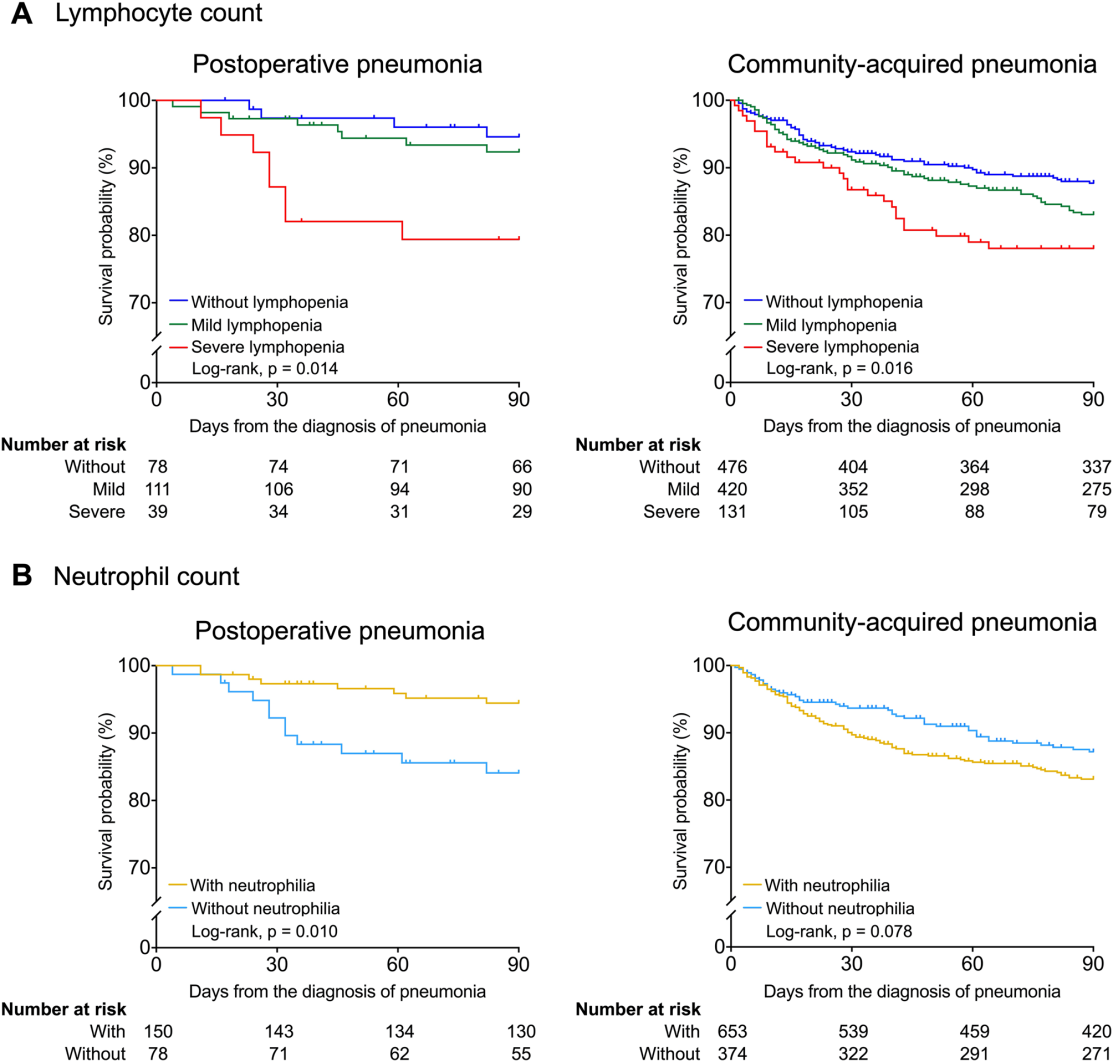
Survival curves for 90-day survival according to lymphocyte and neutrophil counts at pneumonia diagnosis. Kaplan–Meier curves for 90-day survival in patients with postoperative pneumonia and in those with community-acquired pneumonia are shown stratified by absolute lymphocyte count (ALC) (**A**) and neutrophil count (ANC) (**B**) at the time of pneumonia diagnosis. Patients were classified according to ALC as follows: severe lymphopenia, ALC < 0.5 × 10^3^ cells/μL; mild lymphopenia, ALC from 0.5 to 0.9 × 10^3^ cells/μL; and without lymphopenia, ALC ≥ 1.0 × 10^3^ cells/μL. According to ANC, patients with ANC > 7.5 × 10^3^ cells/μL were defined as those with neutrophilia, and patients with ANC ≤ 7.5 × 10^3^ cells/μL were defined as those without neutrophilia. The *P* values were calculated using the log-rank test.

Figure 2A shows the survival curves stratified by ALC at pneumonia diagnosis. In patients with POP, there were significant differences in the survival rate among the three ALC categories (log-rank *p* = 0.014), and the survival rate was worse in the severe lymphopenia group than in the other two groups. In the patients with CAP, severe lymphopenia was also associated with worse 90-day survival (*p* = 0.016). Figure 2B graphically shows curves according to the presence of neutrophilia at the diagnosis of pneumonia. In the patients with POP, neutrophilia at pneumonia diagnosis was associated with better survival (*p* = 0.010). In contrast, CAP patients with neutrophilia tended to have a lower 90-day survival rate (*p* = 0.078).

To estimate the effect of ALC and ANC at pneumonia diagnosis on the all-cause mortality, we additionally performed multivariable analyses, adjusting age, sex, surgery severity score, and SOFA score as clinically important confounders. The findings are shown in Supplementary Table S2. Because of the difference in survival curves between patients with and without severe lymphopenia (Fig. 2A), we compared the effects of severe lymphopenia and no or mild lymphopenia on mortality. In the patients with POP, severe lymphopenia increased the risk of mortality [adjusted HR (aHR), 2.65; 95% CI, 1.00–7.02; *p* = 0.050]. Neutrophilia showed a trend toward decreased mortality risk (aHR, 0.44; 95% CI, 0.17–1.18; *p* = 0.103) in this model. In the patients with CAP, severe lymphopenia and neutrophilia were associated with an increased mortality risk (aHR, 1.39; 95% CI, 0.91–2.12; *p* = 0.129; aHR, 1.41; 95% CI, 0.99–2.01; *p* = 0.059, respectively) although the difference was not statistically significant.

As a sub-analysis, we assessed the effect of ALC and ANC at pneumonia diagnosis on 90-day pneumonia-related mortality. Severe lymphopenia increased the risk of mortality (aHR, 3.66; 95% CI, 1.10–12.19; p = 0.034) in the patients with POP (Supplementary Table S3). The trend of findings was similar to that of the main analysis. For patients who received appropriate initial antibiotic treatment, the survival curves stratified by ALC at pneumonia diagnosis were shown in Supplementary Fig. S1. Key trends were increased risk of mortality from severe lymphopenia and decreased risk of mortality from neutrophilia. These findings were similar to those for all patients with POP. In addition, effects of ALC and ANC at the time of pneumonia diagnosis were also similar for both the 90-day all-cause and pneumonia-related mortality outcomes (Supplementary Tables S4 and S5).

### Time courses of the lymphocyte and neutrophil counts and their effects on survival

**Figure 3:**
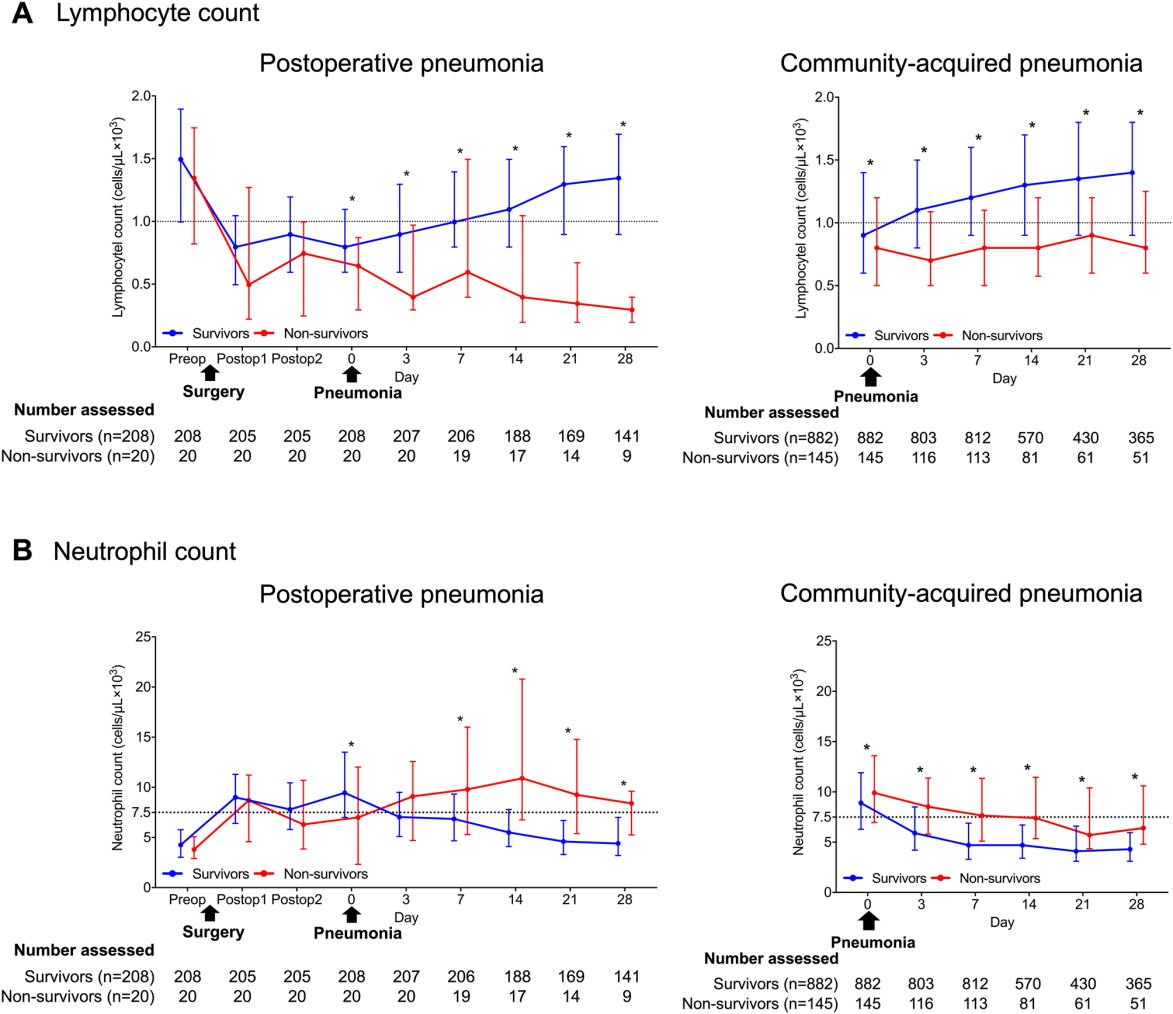
Time courses of absolute lymphocyte and neutrophil counts between survivors and non-survivors. Abbreviation: Preop = Preoperative day, Postop = Postoperative day.Time courses of absolute lymphocyte count (ALC) (**A**) and absolute neutrophil count (ANC) (**B**) between 90-day survivors and non-survivors in postoperative and community-acquired pneumonia are shown. Patients with community-acquired pneumonia included eligible patients without immunocompromised disorders (n = 1027). The dates of the preoperative laboratory test and the first and last postoperative laboratory test before pneumonia diagnosis are expressed as preoperative day, postoperative day 1, and postoperative day 2, respectively. The date of pneumonia diagnosis is described as day 0. The data at each time point are presented as the median and interquartile range. Dotted lines indicate the lower limit of the normal of ALC (1.0 × 10^3^ cells/μL) and the upper limit of the normal of ANC (7.5 × 10^3^ cells/μL). *P value < 0.05 for the comparison of cell counts between survivors and non-survivors at each time point.

Figure 3 shows the temporal changes in ALC and ANC between the survivors and non-survivors. The ALC findings are shown in Fig. 3A. Among the patients with POP, the non-survivors had slightly lower lymphocyte counts before surgery; the lymphocyte counts of both the survivors and non-survivors declined after surgery. Although most patients in both patient groups showed lymphopenia at pneumonia diagnosis, the ALC level remained higher in the survivors than in the non-survivors. After the diagnosis of pneumonia, the ALC of the survivors improved over time whereas that of the non-survivors declined for 3 days after the pneumonia diagnosis and failed to improve thereafter. Among the patients with CAP, the non-survivors showed persistent lymphopenia after the diagnosis; however, the degree of progressive lymphopenia was less severe than in the patients with POP.

The time courses of ANC related to mortality differed between POP and CAP (Fig. 3B). In the patients with POP, the ANC of the survivors and non-survivors increased after surgery. However, the ANC changes after pneumonia diagnosis differed between the survivors and non-survivors. At the pneumonia diagnosis, most of the survivors showed neutrophilia, which rapidly declined after the diagnosis. In contrast, in the non-survivors, ANC failed to increase at the pneumonia diagnosis and showed a delayed increase after the diagnosis. Among the patients with CAP, neutrophilia at diagnosis was more severe and decline in ANC was slower after the diagnosis in the non-survivors than in the survivors.

We also assessed time-course changes of ALC and ANC between survivors and non-survivors with POP who received appropriate initial antibiotics (Supplementary Fig. S2). The trend of findings was almost identical with that for all patients with POP.

## Discussion

In this study, we evaluated the associations between mortality and status of leukocyte phenotypes (ALC and ANC at pneumonia diagnosis and their time-course changes) in patients with POP and examined the differences in those findings between POP and CAP. The main findings of our study were as follows: (1) lymphopenia and neutrophilia at pneumonia diagnosis provided prognostic information in patients with POP; (2) prolonged lymphopenia and delayed increase in neutrophils were characteristic time-course changes of the non-survivors in the patients with POP; and (3) the prognostic effects and time-course changes of ALC and ANC between the survivors and non-survivors in POP showed different trends from those in CAP.

Previous studies have reported that blood lymphocyte and neutrophil counts in critically ill patients reflect the balance of pro- and anti-inflammatory host immune responses^16,26,27^. Host responses to infections have been well-investigated in sepsis^17,28^. However, evidence regarding pneumonia, except in coronavirus disease 19 (COVID-19), is limited. Host responses to infections differ among infection sites^29,30^. Thus, the findings of sepsis, including various infection types, may not be applied to all patients with pneumonia. In the research on pneumonia, investigation of the differences in host immune responses between HAP and CAP is an important topic because levels of immune impairment following acute or chronic illnesses can be higher in HAP than in CAP. Van Vught et al. compared plasma biomarkers and blood gene expression profiles between HAP and CAP and could not detect any significant differences in host responses^31^. In most patients in their study, HAP developed after medical admission, and the patients had various underlying illnesses. Thus, the generalizability of their results based on different patient populations is unclear. We hypothesized that pneumonia following a surgical insult may provide representative information of host immune responses as a second-hit infection. Thus, we compared the characteristics of POP and CAP in which pneumonia was a first-hit infection.

Blood lymphocyte and neutrophil counts have been increasingly recognized as important risk factors for mortality in patients with pneumonia^10–13^. In patients with CAP, Bermejo–Martin et al. demonstrated that lymphopenia (< 724 cells/μL) was independently associated with 30-day mortality in their multicenter observational study^11^. Several studies have shown that lymphopenia is associated with worse outcomes in patients with COVID-19^32,33^. In particular, patients with COVID-19 and severe lymphopenia (< 500 cells/μL) were at a high risk of death^34,35^. The neutrophil count has also been proposed as a prognostic factor in patients with severe CAP and septic shock^13^. However, to the best of our knowledge, the prognostic effects of ALC and ANC in patients with POP have never been evaluated. In our study, severe lymphopenia at diagnosis of pneumonia increased the mortality risk, whereas neutrophilia was associated with improved survival in patients with POP, even after adjustment for severity of surgery and organ failure. Similar trends of these findings were observed when the analysis was performed in patients with POP who received appropriate initial antibiotics and when the endpoint was changed from all-cause mortality to pneumonia-related mortality. In the analysis of patients with CAP, severe lymphopenia was also associated with an increased mortality risk; however, neutrophilia had a negative effect on survival. The findings in patients with CAP in our study were consistent with those in previous studies^10,11^. Thus, our findings suggest that decreased ALC and non-increasing ANC at diagnosis of pneumonia could be prognostic factors in patients with POP, and their effects on mortality differ from those in patients with CAP.

Consecutive biomarker measurement is useful to improve accuracy for the prediction of mortality risk in patients with pneumonia. Previous studies of patients with CAP showed that serial assessment of C-reactive protein, procalcitonin, or mid regional pro-atrial natriuretic peptide was useful for predicting complicated outcomes^36–38^. In our study, ALC declined within 3 days after pneumonia diagnosis and failed to improve over time in non-survivors of POP and CAP. However, the ALC of survivors recovered in both types of pneumonia. These findings were compatible with those of a previous study involving patients with sepsis in which persistent lymphopenia on the fourth day following diagnosis was more important than initial lymphopenia for prediction of mortality risk^15^. Our findings suggest that lymphocyte depletion after pneumonia diagnosis has a significant effect on mortality in patients with pneumonia and that development of therapeutic approach to prevent and improve progressive lymphopenia is a key strategy for improving their mortality.

Understanding the biological mechanisms underlying our findings can help to elucidate the pathogenesis of pneumonia and develop future treatment strategies for improving patient outcomes. Our finding that a higher proportion of appropriate initial antibiotic treatment for patients with POP who were non-survivors versus survivors suggests the importance of development of new therapeutic strategies other than antibiotic treatment. Bone marrow failure or massive migration of immune cells to lungs could explain the fluctuations in ALC or ANC^39,40^. However, there are several other possible mechanisms. In our study, prolonged lymphopenia was observed in the non-survivors. One possible explanation for this phenomenon is lymphocyte apoptosis. Several studies have shown that infection or surgery can induce extensive apoptosis of lymphocytes as anti-inflammatory processes^15,17,19,28^. Profound lymphocyte depletion in patients with POP may be related to recurrent lymphocyte apoptosis induced by the double hits of surgery and pneumonia. In patients with POP who did not survive, the lack of an increase in the neutrophil count at pneumonia diagnosis was a characteristic finding. We speculate that this finding may reflect excessive anti-inflammatory responses induced by surgical procedures. After acute inflammation, anti-inflammatory cytokines or phagocytosis of apoptotic neutrophils can reduce recruitment or production of neutrophils as counter-regulatory mechanisms to compensate for inflammation^41,42^. Based on these hypotheses, our study findings imply that the higher levels of immunosuppression and blunt pro-inflammatory responses are characteristic immunological phenomena of non-survivors in patients with POP. As a reference, previous animal studies revealed that pro-inflammatory cytokine production after pneumonia onset was reduced in the two-hit infection model (sepsis followed by *P. aeruginosa* pneumonia), compared with that in the one-hit model (*P. aeruginosa* pneumonia only)^43,44^. In contrast, pro-inflammatory responses may be associated with an increased mortality risk in patients with CAP. Therefore, immunotherapies that modulate host immune responses can be a good therapeutic option for both POP and CAP. However, different strategies may be needed for these two types of pneumonia^17,28^. Further studies are warranted to verify these hypotheses and to investigate optimal biomarkers to accurately characterize the immune status of patients with POP, as in recent research for COVID-19^45^.

There were several limitations in this study. First, this was a single-center study, so our findings may not be generalizable to other institutions. Second, our analyses could not fully consider therapeutic factors, including the overuse of broad-spectrum antibiotics, that might affect ALC and ANC kinetics. Third, we could not assess pathogen-specific characteristics because of an insufficiently large sample. Therefore, our findings should be validated in large-scale prospective studies by different research groups. However, we believe that our results provide a basis for future investigations, including immunological research to improve outcomes of patients with pneumonia.

## Conclusions

In patients with POP, ALC and ANC at pneumonia diagnosis was useful for stratifying mortality risks. In addition, the time-course of ALC and ANC differed between survivors and non-survivors in patients with POP. Prolonged lymphopenia and a delayed increase in neutrophils were characteristic findings associated with mortality. These findings showed different trends from those in patients with CAP, suggesting that patients with POP have immunological features distinct from those of patients with CAP. Future studies are warranted to validate the prognostic significance of ALC and ANC and to elucidate the biological mechanisms of our findings for the development of future immunomodulatory therapies.

## Supplementary Information

Supplementary Information.

## Data Availability

The datasets analyzed during the current study are available from the corresponding author on reasonable request.

## Abbreviations

ALC: Absolute lymphocyte count
ANC: Absolute neutrophil count
CAP: Community-acquired pneumonia
CI: Confidence interval
COVID-19: Coronavirus disease 19
HAP: Hospital-acquired pneumonia
HCAP: Health-care associated pneumonia
HR: Hazard ratio
NLR: Neutrophil to lymphocyte ratio
POP: Postoperative pneumonia
SOFA: Sequential organ failure assessment
VAP: Ventilator-associated pneumonia
