# Supplementary material for: Effects of lymphocyte and neutrophil counts and their time courses on mortality in patients with postoperative pneumonia
Source: Sci Rep. 2022 Aug 26;12:14564. doi: 10.1038/s41598-022-18794-5 (PMC9411836; doi:10.1038/s41598-022-18794-5)
Supplement: Supplementary file 1 — Supplementary Information. [file 41598_2022_18794_MOESM1_ESM.docx]

**Supplementary information**

**Supplementary Figure S1.** **Survival curves for 90-day survival according to lymphocyte and neutrophil counts at pneumonia diagnosis in patients with postoperative pneumonia who received appropriate initial antibiotics**


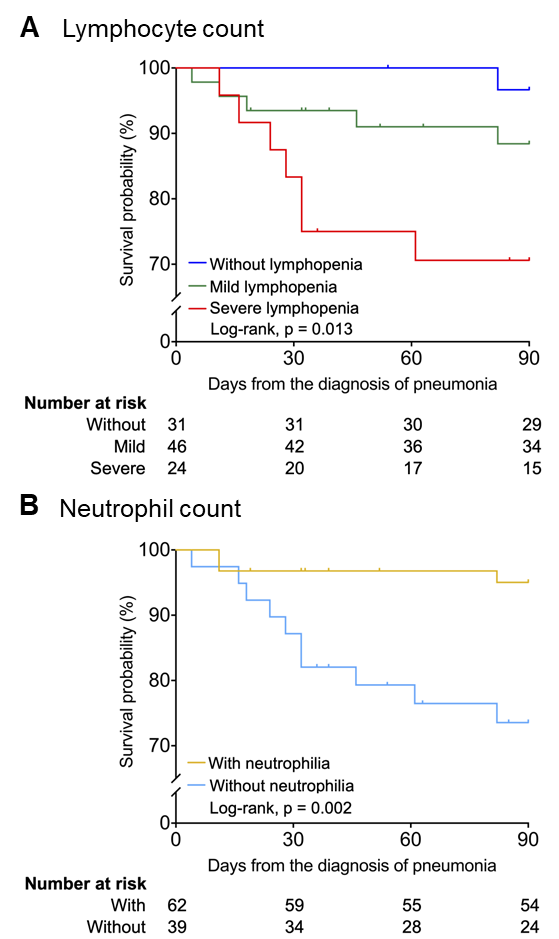


Kaplan–Meier curves for 90-day survival in patients with postoperative pneumonia are shown stratified by absolute lymphocyte count (ALC) (A) and neutrophil count (ANC) (B) at the time of pneumonia diagnosis. Patients who received appropriate initial antibiotics were included in this analysis. Patients were classified according to ALC as follows: severe lymphopenia, ALC < 0.5 × 10^3^ cells/μL; mild lymphopenia, ALC from 0.5 to 0.9 × 10^3^ cells/μL; and without lymphopenia, ALC ≥ 1.0 × 10^3^ cells/μL. According to ANC, patients with ANC > 7.5 × 10^3^ cells/μL were defined as those with neutrophilia, and patients with ANC ≤ 7.5 × 10^3^ cells/μL were defined as those without neutrophilia. The *P* values were calculated by the log-rank test.

**Supplementary Figure S2.** **Time courses of absolute lymphocyte and neutrophil counts between survivors and non-survivors who received appropriate initial antibiotics**


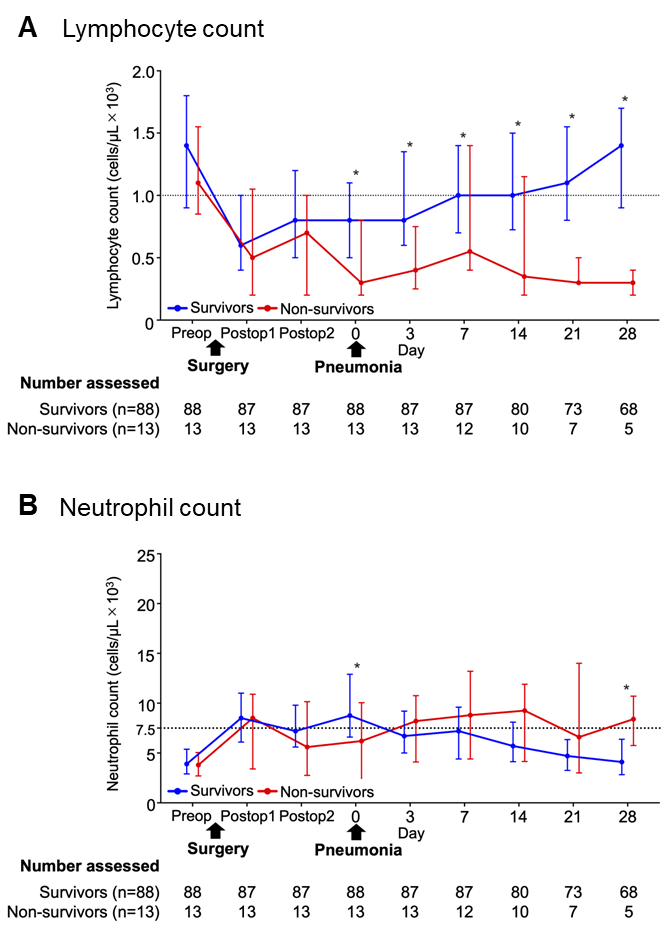


Abbreviation: Preop = Preoperative day, Postop = Postoperative day.

Time courses of absolute lymphocyte count (ALC) (A) and absolute neutrophil count (ANC) (B) between 90-day survivors and non-survivors in postoperative pneumonia who received appropriate initial antibiotics are shown. The dates of the preoperative laboratory test and the first and last postoperative laboratory test before pneumonia diagnosis are expressed as preoperative day, postoperative day 1, and postoperative day 2, respectively. The date of pneumonia diagnosis is described as day 0. The data at each time point are presented as the median and interquartile range. Dotted lines indicate the lower limit of the normal of ALC (1.0 × 10^3^ cells/μL) and the upper limit of the normal of ANC (7.5 × 10^3^ cells/μL).

^*^The *P* value < 0.05 for the comparison of cell counts between survivors and non-survivors at each time point.

**Supplementary Table S1. Identified pathogens in patients with postoperative pneumonia**

| Pathogens | | | Postoperative pneumonia |
| --- | --- | --- | --- |
|  |  |  | (n = 228) |
| Identified | | | 155 (68.0) |
|  | Gram-positive pathogens | |  |
|  |  | *Staphylococcus aureus* (MRSA) | 20 (8.8) |
|  |  | *S. aureus* (MSSA) | 13 (5.7) |
|  |  | *Enterococcus* spp. | 12 (5.3) |
|  |  | *Streptococcus pneumoniae* | 8 (3.5) |
|  |  | Other gram-positive pathogens | 2 (0.9) |
|  | Gram-negative pathogens | |  |
|  |  | *Enterobacter* spp. | 39 (17.1) |
|  |  | *Pseudomonas aeruginosa* | 37 (16.2) |
|  |  | *Klebsiella pneumoniae* | 28 (12.3) |
|  |  | *Acinetobacter* spp. | 17 (7.5) |
|  |  | *Escherichia coli* | 15 (6.6) |
|  |  | *Haemophilus influenzae* | 13 (5.7) |
|  |  | *Stenotrophomonas maltophilia* | 9 (3.9) |
|  |  | *Serratia marcescens* | 8 (3.5) |
|  |  | *Citrobacter* spp. | 4 (1.8) |
|  |  | *Klebsiella oxytoca* | 4 (1.8) |
|  |  | *Moraxella catarrhalis* | 3 (1.3) |
|  |  | Other gram-negative pathogens | 11 (4.8) |
| Unidentified | | | 73 (32.0) |

Abbreviations: MRSA = methicillin-resistant *Staphylococcus aureus*; MSSA = methicillin-susceptible *Staphylococcus aureus*.

Data are presented as n (%) unless indicated otherwise.

**Supplementary Table S2. Multivariate analysis to assess the all-cause mortality risks of lymphocyte and neutrophil counts at pneumonia diagnosis**

| **A. Postoperative pneumonia** | | |  | | |
| --- | --- | --- | --- | --- | --- |
| Category | Variable | | Postoperative pneumonia | | |
|  |  |  | Adjusted HR | 95% CI | *P* value |
| Age (years) | Age ≥ 70^*^ | | 0.86 | 0.35–2.11 | 0.737 |
| Sex | Female | | 2.03 | 0.70–5.88 | 0.192 |
| Lymphocyte count | Severe lymphopenia^†^ | | 2.65 | 1.00–7.02 | 0.050 |
| Neutrophil count | With neutrophilia^‡^ | | 0.44 | 0.17–1.18 | 0.103 |
| Severity of surgery | Severity score ≥ 23^*^ | | 2.42 | 0.82–7.16 | 0.111 |
| Severity of illness | SOFA score ≥ 9^*^ | | 1.81 | 0.71–4.61 | 0.213 |
|  |  |  |  |  |  |
| **B. Community-acquired pneumonia** | | |  |  |  |
| Category | Variable | | Community-acquired pneumonia | | |
|  |  |  | Adjusted HR | 95% CI | *P* value |
| Age (years) | Age ≥ 75^*^ | | 1.18 | 0.83–1.68 | 0.354 |
| Sex | Female | | 0.68 | 0.45–1.03 | 0.066 |
| Lymphocyte count | Severe lymphopenia^†^ | | 1.39 | 0.91–2.12 | 0.129 |
| Neutrophil count | With neutrophilia^‡^ | | 1.41 | 0.99–2.01 | 0.059 |
| Severity of illness | Pneumonia Severity Index class | |  |  |  |
|  |  | I–III | 1 (Ref) | | |
|  |  | IV | 2.21 | 1.40–3.49 | 0.001 |
|  |  | V | 3.77 | 2.39–5.96 | < 0.001 |

Abbreviations: SOFA = Sequential Organ Failure Assessment; PSI = Pneumonia Severity Index; HR = hazard ratio; CI = confidence interval. All-cause mortality risks of patients with postoperative pneumonia (A) and community-acquired pneumonia (B) were calculated using the Cox proportional hazards model.

^*^When selecting the cutoff values of these continuous variables, the values from previous studies and the data distribution of each variable in this study were considered.

^†^According to absolute lymphocyte counts (ALC), patients with ALC of < 0.5 × 10^3^ cells/μL were defined as those with severe lymphopenia, and patients with ALC ≥ 0.5 × 10^3^ cells/μL were defined as no or mild lymphopenia.

^‡^According to absolute neutrophil counts (ANC), patients with ANC of > 7.5 × 10^3^ cells/μL were defined as having neutrophilia, and patients with ANC ≤ 7.5 × 10^3^ cells/μL were defined as without neutrophilia.

**Supplementary Table S3. Univariate and multivariate analyses to assess pneumonia-related mortality risks of lymphocyte and neutrophil counts at pneumonia diagnosis**

| **A. Univariate analysis** | | |  | | |
| --- | --- | --- | --- | --- | --- |
| Category | Variable | | Crude HR | 95% CI | *P* value |
| Lymphocyte count | Severe lymphopenia^*^ | | 5.11 | 1.65–15.85 | 0.005 |
| Neutrophil count | With neutrophilia^†^ | | 0.36 | 0.11–1.13 | 0.079 |
|  |  |  |  |  |  |
| **B. Multivariate analysis** | | |  | | |
| Category | Variable | | Adjusted HR | 95% CI | *P* value |
| Age (years) | Age ≥ 70^‡^ | | 2.16 | 0.57–8.14 | 0.256 |
| Sex | Female | | 2.39 | 0.63–9.08 | 0.201 |
| Lymphocyte count | Severe lymphopenia^*^ | | 3.66 | 1.10–12.19 | 0.034 |
| Neutrophil count | With neutrophilia^†^ | | 0.56 | 0.16–1.95 | 0.364 |
| Severity of surgery | Severity score ≥ 23^‡^ | | 0.79 | 0.10–6.53 | 0.826 |
| Severity of illness | SOFA score ≥ 9^‡^ | | 2.11 | 0.64–6.94 | 0.218 |

Abbreviations: SOFA = Sequential Organ Failure Assessment; HR = hazard ratio; CI = confidence interval.

The risk of pneumonia-related mortality in patients with postoperative pneumonia was calculated using the Cox proportional hazards model. Pneumonia-related mortality was defined as death caused by respiratory failure or multiple organ failure following postoperative pneumonia.

^*^According to absolute lymphocyte count (ALC), patients with ALC of < 0.5 × 10^3^ cells/μL were defined as those with severe lymphopenia, and patients with ALC ≥ 0.5 × 10^3^ cells/μL were defined as no or mild lymphopenia.

^†^According to absolute neutrophil counts (ANC), patients with ANC of > 7.5 × 10^3^ cells/μL were defined as having neutrophilia, and patients with ANC ≤ 7.5 × 10^3^ cells/μL were defined as without neutrophilia.

^‡^When selecting the cutoff values of these continuous variables, the values from previous studies and the data distribution of each variable in this study were considered.

**Supplementary Table S4.** **Univariate and multivariate analyses to assess all-cause mortality risks of lymphocyte and neutrophil counts at pneumonia diagnosis in patients who received appropriate initial antibiotics**

| **A. Univariate analysis** | | |  |  |  |
| --- | --- | --- | --- | --- | --- |
| Category | Variable | | Crude HR | 95% CI | *P* value |
| Lymphocyte count | Severe lymphopenia^*^ | | 4.15 | 1.39–12.35 | 0.011 |
| Neutrophil count | With neutrophilia^†^ | | 0.17 | 0.05–0.62 | 0.007 |
|  |  |  |  |  |  |
| **B. Multivariate analysis** | | |  |  |  |
| Category | Variable | | Adjusted HR | 95% CI | *P* value |
| Age (years) | Age ≥ 70^‡^ | | 0.96 | 0.31–3.01 | 0.942 |
| Sex | Female | | 2.68 | 0.66–10.87 | 0.169 |
| Lymphocyte count | Severe lymphopenia^*^ | | 2.45 | 0.75–8.03 | 0.140 |
| Neutrophil count | With neutrophilia^†^ | | 0.21 | 0.05–0.97 | 0.045 |
| Severity of surgery | Severity score ≥ 23^‡^ | | 2.19 | 0.54–8.87 | 0.273 |
| Severity of illness | SOFA score ≥ 9^‡^ | | 1.44 | 0.41–5.07 | 0.573 |

Abbreviations: SOFA = sequential organ failure assessment; HR = hazard ratio; CI = confidence interval.

The risk of all-cause mortality in patients with postoperative pneumonia was calculated using the Cox proportional hazards model. Patients who received appropriate initial antibiotics (n = 101) were included in this analysis, and all-cause death occurred in 13.

^*^According to absolute lymphocyte counts (ALC), patients with ALC of < 0.5 × 10^3^ cells/μL were defined as those with severe lymphopenia, and patients with ALC ≥ 0.5 × 10^3^ cells/μL were defined as no or mild lymphopenia.

^†^According to absolute neutrophil counts (ANC), patients with ANC of > 7.5 × 10^3^ cells/μL were defined as having neutrophilia, and patients with ANC ≤ 7.5 × 10^3^ cells/μL were defined as without neutrophilia.

^‡^When selecting the cutoff values of these continuous variables, the values from previous studies and the data distribution of each variable in this study were considered.

**Supplementary Table S5.** **Univariate and multivariate analyses to assess the pneumonia-related mortality risks of lymphocyte and neutrophil counts at pneumonia diagnosis in patients who received appropriate initial antibiotics**

| **A. Univariate analysis** | | |  |  |  |
| --- | --- | --- | --- | --- | --- |
| Category | Variable | | Crude HR | 95% CI | *P* value |
| Lymphocyte count | Severe lymphopenia^*^ | | 8.55 | 1.66–44.12 | 0.010 |
| Neutrophil count | With neutrophilia^†^ | | 0.24 | 0.05–1.22 | 0.085 |
|  |  |  |  |  |  |
| **B. Multivariate analysis** | | |  |  |  |
| Category | Variable | | Adjusted HR | 95% CI | *P* value |
| Age (years) | Age ≥ 70^‡^ | | 2.53 | 0.44–14.65 | 0.300 |
| Sex | Female | | 3.97 | 0.63–25.24 | 0.144 |
| Lymphocyte count | Severe lymphopenia^*^ | | 5.97 | 1.05–33.83 | 0.043 |
| Neutrophil count | With neutrophilia^†^ | | 0.37 | 0.05–2.90 | 0.034 |
| Severity of surgery | Severity score ≥ 23^‡^ | | 1.33 | 0.14–12.34 | 0.800 |
| Severity of illness | SOFA score ≥ 9^‡^ | | 2.40 | 0.37–15.56 | 0.360 |

Abbreviations: SOFA = sequential organ failure assessment; HR = hazard ratio; CI = confidence interval.

The risk of pneumonia-related mortality in patients with postoperative pneumonia was calculated using the Cox proportional hazards model. Patients who received appropriate initial antibiotics (n = 101) were included in this analysis, and pneumonia-related death occurred in 7.

^*^According to absolute lymphocyte counts (ALC), patients with ALC of < 0.5 × 10^3^ cells/μL were defined as those with severe lymphopenia, and patients with ALC ≥ 0.5 × 10^3^ cells/μL were defined as no or mild lymphopenia.

^†^According to absolute neutrophil counts (ANC), patients with ANC of > 7.5 × 10^3^ cells/μL were defined as having neutrophilia, and patients with ANC ≤ 7.5 × 10^3^ cells/μL were defined as without neutrophilia.

^‡^When selecting the cutoff values of these continuous variables, the values from previous studies and the data distribution of each variable in this study were considered.
